# Design and Verification of a Non-Contact Body Dimension Measurement System for Jiangquan Black Pigs Based on Dual-View Depth Vision

**DOI:** 10.3390/ani15243601

**Published:** 2025-12-15

**Authors:** Zhao Ma, Shiyin Li, Zhanchi Ren, Jing Wang, Junfeng Chen, Wei Chen, Hui Tang, Yarui Gao, Yunpeng Li, Baosong Xing, Yongqing Zeng

**Affiliations:** 1 Shandong Provincial Key Laboratory for Livestock Germplasm Innovation & Utilization, College of Animal Science and Technology, Shandong Agricultural University, Taian 271018, China; 2 Henan Key Laboratory of Farm Animal Breeding and Nutritional Regulation, Henan Pig Breeding Engineering Research Centre, Institute of Animal Husbandry, Henan Academy of Agricultural Sciences, Zhengzhou 450002, China

**Keywords:** non-contact measurement, depth camera, dual-view, body dimension measurement, Jiangquan black pig

## Abstract

To address inefficiencies, pig stress from traditional manual body dimension measurement, and environmental interference in existing automated technologies, this study designed and validated a non-contact measurement system for Jiangquan black pigs based on dual-view (top + side) depth vision (Intel RealSense D455). Key dimensions (body length/width/height, chest depth) were accurately extracted via depth map calibration, dynamic scaling, and U-Net segmentation. Chest girth was estimated using the Ramanujan ellipse perimeter model (MAE = 4.15 cm, R^2^ = 0.908) and integrated as the core parameter for body weight prediction in an empirical formula. This experimental dataset comprises 30 pigs sourced from a single farm, with body weights falling within a limited range (30–100 kg). All dimensions achieved R^2^ > 0.9, with top-view body width performing best (R^2^ = 0.9424, MAE = 1.9 cm). Body weight prediction yielded R^2^ = 0.957 and MAE = 5.1 kg. The system completes measurements in 24 ± 4 s with low hardware costs and stress-free operation, making it suitable for precision breeding in small-to-medium pig farms.

## Simple Summary

To overcome the low efficiency, high cost, and susceptibility to environmental interference of traditional pig body measurement methods and existing automation technologies, this study designed and validated a non-contact body measurement system for Jiangquan Black pigs based on dual-view depth vision. Employing a “top-view + side-view” collaborative acquisition framework, the system accurately extracts body measurements such as length, width, and height through depth map calibration and dynamic scaling techniques, while estimating chest circumference using an elliptical model. Validation experiments on 30 Jiangquan Black pigs demonstrated high consistency between the system and manual measurements. The weight prediction model achieved an R^2^ value of 0.957, with each measurement taking only 24 ± 4 s. This efficient, low-cost, and stress-free system is suitable for precision breeding management in small-to-medium-sized pig farms.

## 1. Introduction

In contemporary swine production, meticulous observation of pig body conformation and weight is paramount to achieving efficient economic management and safeguarding animal health. Abnormal fluctuations in these indicators frequently function as early warning signals for potential diseases [1,2]. Consequently, the regular and accurate acquisition of pertinent data is imperative for sustaining optimal herd health. Moreover, it provides a scientific foundation for optimizing reproductive performance and enhancing production efficiency [3]. Conventional measurement techniques predominantly depend on manual, contact-based procedures: Caretakers utilize tools such as tape measures and measuring rods to manually record body length, width, and other dimensions, while weighing scales are employed to document body weight. However, these approaches have exhibited substantial limitations: The implementation of manual operations engenders considerable measurement errors, thereby compromising the accuracy of the resulting data. Conversely, the process of herding and restraining pigs instigates profound stress responses, exerting a deleterious effect on growth performance, constituting a violation of animal welfare standards, and augmenting the physical burden on farm workers [4]. In recent years, the livestock industry has witnessed a marked shift in its approach to measurement technologies, with a rapid transition toward intelligent and digital transformation. This transition, characterized by the accelerated adoption of non-contact automated measurement methods, signifies a significant departure from the conventional manual measurement techniques that have historically dominated the industry [5,6]. In light of these developments, image-based methods for estimating pig weight and body conformation have emerged as a research focus, owing to their non-contact and high-efficiency characteristics [7,8,9]. The automated and precise acquisition of growth parameters through such technologies enables farms to implement refined feeding management, ultimately achieving cost reduction, efficiency gains, and enhanced overall economic benefits [10,11].

At present, image-based methods for estimating pig weight and body conformation can be broadly categorized into six types:

− Projection methods relied on grids and stereoscopic projection principles for calculations. However, the efficacy of these methods is contingent upon the utilization of specialized equipment and the execution of manual interventions, which engenders challenges related to automation [12].
− The 2D image-based methods employ the extraction of parameters such as contours and projected area from 2D images of the pig’s back to construct weight estimation models [13]. However, this approach is characterized by its suboptimal accuracy and stringent requirements for pig posture [14].
− 3D imaging method: The utilization of depth cameras to capture three-dimensional point cloud data of the pig’s back facilitates the extraction of multidimensional feature parameters, including height. These parameters exhibit strong correlations with weight and body conformation, demonstrating significant application potential. This approach has been demonstrated to yield superior mean absolute error (MAE) performance in body conformation estimation [15,16,17].
− Ellipse fitting method: As an optimized approach to the 2D image method, it standardizes shape parameter extraction through ellipse modeling, achieving an average relative error (ARE) between 3% and 3.8% [18,19].
− Deep learning-driven image feature fusion methods utilize models like CNNs and Transformers to automatically extract high-dimensional semantic features and fuse multimodal information (e.g., RGB images + texture features, local regions + global morphological features) to construct weight estimation models, eliminating the need for manual feature design [20,21,22].
− Multi-view image fusion weight estimation methods combine multi-angle image data (e.g., top-view + side-view, front-view + side-view) to compensate for single-view information limitations through registration and feature fusion, constructing more comprehensive body shape representation models [23,24,25,26].

Despite the presence of distinct technical merits, the majority of extant methods necessitate the undertaking of cumbersome preprocessing (e.g., background removal, enhancement, binarisation, filtering, head/tail trimming) that is both time-consuming and susceptible to environmental interference (e.g., uneven lighting, obstructions), thus hindering the development of fully automated workflows [12]. Consequently, the development of an automated pig weight and body conformation estimation method with minimal manual intervention, simplified preprocessing, high accuracy, and robustness is imperative for the intelligent pig farming industry. To address this need, this study proposes a deep learning-based weight estimation method that extracts key features from pig body images to reduce reliance on complex preprocessing. The proposed system integrates 3D vision, 2D image analysis, ellipse modeling, deep learning segmentation, and multi-view fusion—not a mere aggregation of technologies—forming a systematic, automated, and high-precision non-contact measurement solution with both innovation and practical value. This solution is representative of “multi-view fusion + 3D The objective of the cross-disciplinary integration of “vision + ellipse modeling” is to provide an innovative technical solution for the automated and precise monitoring of pig growth information.

## 2. Materials and Methods

This study was conducted in July 2024 at a commercial pig farm operated by Shandong Futong Agriculture and Animal Husbandry Development Co., Ltd. (Linyi, China). The study had certain limitations, with a total of 30 healthy Jiangquan Black pigs in the finishing stage (60–180 days old, weighing 30–100 kg) selected as experimental subjects. Prior to the experiment, all animals underwent veterinary examination to exclude individuals with physical defects such as limb deformities or injuries. The pigs were housed in a standardized environment equipped with automatic climate control, automatic feeding systems, and automatic watering systems. One week before data collection, all test pigs were transitioned to a uniform feeding regimen (complete formulated feed, 2 kg per portion, administered three times daily) to minimize dietary influence on body conformation.

### 2.1. Reference Manual Measurements

**Figure 1:**
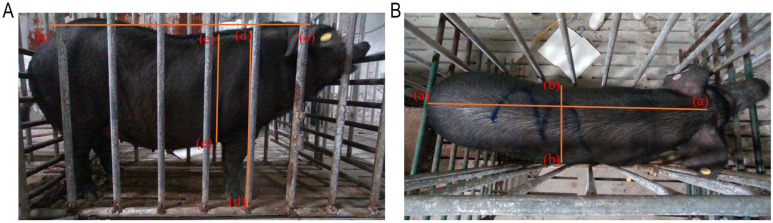
Schematic diagram illustrating key body measurement locations for body length, body width, chest depth, and body height in pigs. (**A**) Side view, where the distance between the two points (d) represents the body height, the distance between the two points (e) represents the chest depth, and the distance between the two points (c) represents the body length; (**B**) Top view, where the distance between the two points (a) represents the body length, and the distance between the two points (b) represents the body width.

All manual measurements were conducted with the assistance of two experienced technicians (each with over five years of professional experience) in strict adherence to standardized procedures, using a measuring tape with an accuracy of ≤1 mm. Each parameter was measured three consecutive times, and the average value was adopted as the final measurement result. The measurement process is illustrated in Figure 1:

− Body Length (BL): Distance from the midpoint between the ears to the tail root.
− Body Width (BW): Measured at the maximum abdominal width.
− Body Height (BH): Vertical distance from the scapular summit (withers) to the ground.
− Chest Depth(CD): is defined as the vertical distance from the highest point of the withers to the lowest point of the sternum behind the shoulder blades.
− Bust: Using a tape measure with an accuracy of 0.1 cm, encircle the pig’s body at the intersection point between the posterior edge of the scapula and the widest part of the thoracic cage. Keep the tape measure horizontal and snug against the skin, avoiding compression of soft tissue.

### 2.2. Hardware System and Data Acquisition

**Figure 2:**
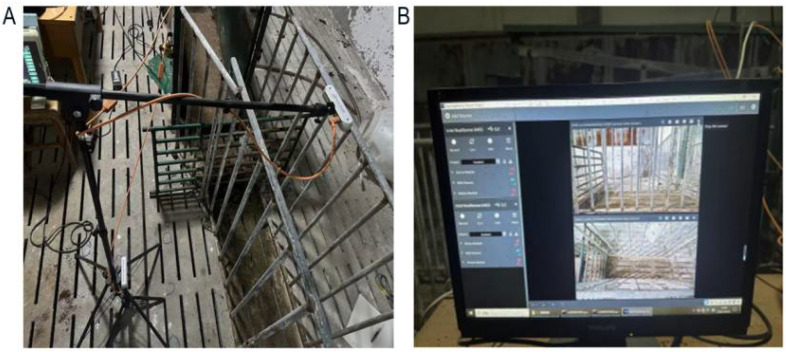
Schematic diagram of the experimental setup in the pig barn, showing positions of top-view and side-view cameras relative to the measurement zone. (**A**) Schematic diagram of the experimental setup in the pig pen. (**B**) Display monitor.

The core hardware of this automated measurement system employs the Intel RealSense D455 depth camera (RealSense, Santa Clara, CA, USA), selected for its portability and a depth error specification of ≤2 mm. Dynamic calibration is required prior to use: install the latest version of the RealSense SDK 2.0 and Viewer tool (V2.55.1), and place the Dynamic-Calibration Tool on a wall 0.6–0.85 m away from the calibration board. After connecting the camera, operate through the “Chip Calibration” function in the “Depth Tools” module of the Viewer software (V2.55.1). Move the camera to ensure the calibration board covers the field of view at different angles. Before saving the parameters, verify that the depth image fill rate is ≥99% and the error is ≤2%. The same calibration method was applied to both cameras. No pigs are allowed during camera calibration. The cameras were connected to a standard laptop equipped with a 12th-generation Intel Core i5-12450H processor (16 GB RAM) (Intel, Shanghai, China), running a customized PyQt5 data processing software (5.15.11). For dual-view acquisition, the cameras were mounted on adjustable tripods (Figure 2).

The data collection process was conducted within the confines of the pig house, utilizing the ambient lighting present to mitigate the introduction of stress or depth noise from extraneous light sources. The subjects (i.e., the pigs) were guided to a dedicated, flat measurement zone at the exit of the passageway. The system operated in two modes:

Top-View Mode: Camera positioned 140 cm vertically above the center of the measurement zone.

Side-View Mode: Camera placed 70 cm laterally from the side edge of the zone.

RGB images and depth images for each pig were captured at a resolution of 848 × 480 pixels and a frame rate of 15 frames per second. Three sets of images were collected per pig, with a single operator selecting the set exhibiting the clearest body contour and most natural posture (no struggling, no arching of the back) for subsequent analysis.

### 2.3. Image Pre-Processing and Segmentation

Depth Image Processing: Raw 16-bit depth maps (unit: mm) from the D455 camera were converted to metric units using scale factor:

$$k_{\mathrm{scale}}=0.001,i.e.,{\mathrm{depth}}_{m}={\mathrm{depth}}_{16}\times k_{\mathrm{scale}}$$

For visualization, depth maps were contrast-enhanced and converted to pseudo-color images using the Jet colormap.

**Figure 3:**
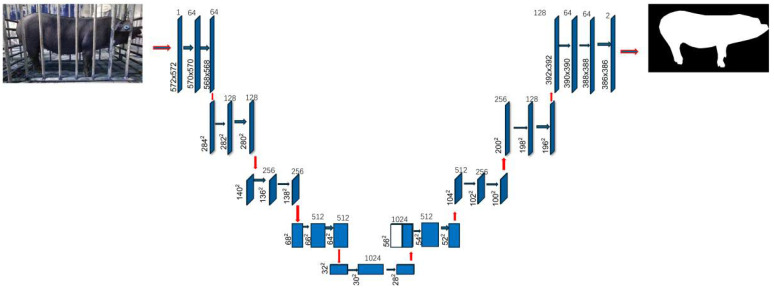
Architecture of the U-Net network used for pig body segmentation in side-view.

Side-View Image Segmentation: To eliminate background interference (e.g., fences, ground) in side-view RGB images, a U-Net semantic segmentation model was employed for precise extraction of the pig body region (Figure 3). A total of 150 lateral-view RGB images of pigs in pen environments were manually annotated using LabelMe 4.5.13 software. The annotated dataset was randomly split into a training set (105 images) and a validation set (45 images) at a 7:3 ratio to avoid model overfitting. The U-Net model was trained for 50 epochs with the Adam optimizer (learning rate = 1 × 10^−4^, batch size = 2), and its segmentation performance was evaluated using Intersection over Union (IoU) and Dice Similarity Coefficient (DSC). The final validation set achieved an IoU of 0.90 ± 0.05 and a DSC of 0.94 ± 0.04. This model effectively distinguishes pigs from complex barn backgrounds and outputs clean binary contour maps, laying a foundation for subsequent measurement tasks [21,22].

### 2.4. Acquisition of Body Dimensions and Automated Weight Estimation

**Figure 4:**
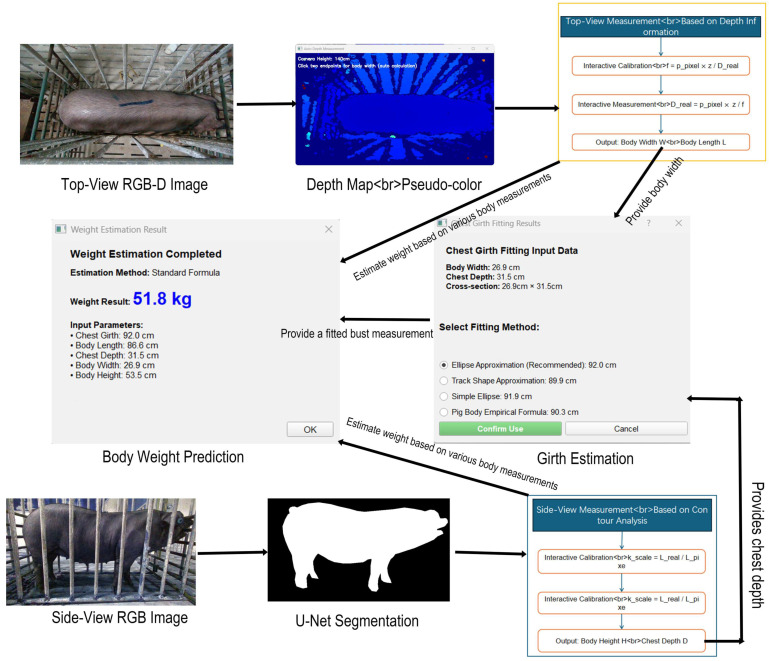
Workflow of body dimension acquisition and body weight estimation.

The objective of this study was to develop a non-contact automated measurement method for the estimation of body dimensions and weight in Jiangquan black pigs. To this end, an integrated pipeline was established, combining top-view and side-view data. The process commences with the extraction of key body size parameters from top-view and side-view images using depth vision and image segmentation techniques, respectively. Subsequently, the chest circumference is estimated based on an elliptical model, and finally, body weight is accurately predicted through the fusion of multiple empirical formulas. The overall algorithm workflow is illustrated in Figure 4.

Top-View Measurement: This process is based on depth information. First, the user calibrates the system by marking a line segment of known physical length on the top-view depth map to calculate the effective focal length f. Subsequently, by interactively selecting the endpoints for body width and body length on the same image, the system converts the pixel distance into real-world length based on the pinhole camera model and depth information, directly outputting the Body Width (W) and Body Length (L). The focal length calibration employs the following formula:

$$f=\frac{p_{pixel}\times z}{D_{real}}$$

The real-distance calculation for body dimensions then uses:

$$D_{real}\frac{p_{pixel}\times z}{f}\times 100$$

$D_{real}$: The actual physical length of the pig body target dimension (unit: cm), i.e., the final output body length L or body width W.
$p_{pixel}$: length of the pig’s target dimensions (body length/body width) (unit: pixel), calculated by the difference in pixel coordinates between the selected endpoints.
Z: The average depth value (in meters) of the region corresponding to the pig’s target dimension is obtained by statistical analysis of the depth data from the corresponding area in the depth map.
f: Step 1 The effective focal length of the camera obtained through calibration (unit: pixels).

Side-View Measurement: This process is based on contour analysis. The side-view RGB image is first processed by a U-Net model for semantic segmentation, generating a binary contour map that eliminates background interference. The user then calibrates the scale by marking a known length on this contour map to determine the scale factor $k_{scale}$. By interactively selecting key points for body height and chest depth, the system converts pixel distances proportionally, outputting the Body Height (H) and Chest Depth (D). The scale factor is determined as:

$$k_{scale}=\frac{L_{real}}{L_{pixel}}$$

The physical dimension calculation is performed using:

$$L_{physical\;}=L_{pixel}\times k_{scale}$$

Girth Estimation: To address the challenge of directly measuring pig chest circumference, this study modeled the cross-section of the pig’s chest as an elliptical structure. The top-view Body Width (W) serves as the major axis, and the side-view Chest Depth (D) serves as the minor axis. The fitted Chest Girth (C) is calculated using Ramanujan’s approximation for the ellipse perimeter, which maintains high accuracy across a wide range of ellipticities. The formula is defined as:

$$C=\pi \left[3(a+b)-\sqrt{\;(3a+b)(a+3b)}\right]$$

where a = W/2 (semi-major axis) and b = D/2(semi-minor axis).

Body Weight Prediction: This study adopted an integrated approach based on empirical formulas for body weight prediction, rather than relying on complex machine learning models, to enhance model interpretability and suitability for environments with limited computational resources. The system integrated several calibrated classical weight estimation formulas and fused the optimal results via linear regression. Core body weight estimation formulas include:

$$BW_{1}=0.634\times \frac{C^{2}\times L}{12500}+14.6\;(\mathrm{Standard}\;\mathrm{Formula})$$

$$BW_{2}=1.247\times \frac{C\times L\times D}{7500}+7.4$$

$$BW_{3}=2.142\times \frac{W\times D\times L}{7200}+6.9$$

$$BW_{4}=1.245\times \frac{\left(C\times L\times D\times 0.8\right)+\left(H\times 50\right)}{1000}+7.4$$

where C is the fitted abdominal girth, L is the top-view body length, W is the top-view body width, D is the side-view chest depth, and H is the side-view body height.

The final weight was determined using a linear regression model integrating key empirical formulas, with the linear fit equation being $\mathrm{BW}=\;0.8294\times {\mathrm{BW}}_{1}+\;11.16$, predominantly driven by the standard formula ${\mathrm{BW}}_{1}$.

Validation Method: Evaluate model generalization using 5-fold cross-validation: Randomly partition the dataset into 30 groups and divide them into 5 mutually exclusive subsets (6 groups per fold). For each iteration, use 4 folds as the training set to fit the regression equation, while the remaining 1 fold serves as the validation set to assess performance. Repeat this process 5 times and calculate the average result. The cross-validation average R^2^ =0.949.

Overfitting Risk Assessment: With a sample size of (n = 30), the calibration model in this study is a single-variable linear model (only 2 parameters), whose complexity is far below the fitting capability corresponding to the sample size (typically, linear models require a sample size ≥ 5 × number of parameters; in this study, (30 ≥ 10)). Combined with 5-fold cross-validation results, The difference in (R^2^) between the training and validation sets is only 0.008, indicating no significant performance degradation. Therefore, the risk of overfitting is extremely low.

### 2.5. Statistical Analysis and Model Validation

The performance of the system was evaluated using a dataset comprising 30 pigs. The measurement results from the automated system were compared with manual reference measurements (ground truth), employing Mean Absolute Error (MAE) and Root Mean Square Error (RMSE) as evaluation metrics. For the weight estimation model, its predictive performance was assessed based on the coefficient of determination (R^2^), MAE, RMSE, and Mean Absolute Percentage Error (MAPE). All aforementioned metrics were calculated using the same dataset to ensure consistency [27].

$$R^{2}=1-\frac{\frac{1}{n}\sum_{i=1}^{n}{\left(y_{i}-\;{\hat{y}}_{i}\right)}^{2}}{\frac{1}{n}\sum_{i=1}^{n}{\left(y_{i}-\;\overset{-}{y}\right)}^{2}}$$

$$\mathrm{MAE}\;=\frac{1}{n}\sum_{i=1}^{n}\left|y_{i}-\;{\hat{y}}_{i}\right|$$

$$\mathrm{MAPE}=\frac{1}{n}\sum_{i=1}^{n}\left|\frac{y_{i}-\;{\hat{y}}_{i}}{y_{i}}\right|\times 100$$

$$RMSE=\sqrt{\frac{1}{n}\sum_{i=1}^{n}{\left(y_{i}-\;{\hat{y}}_{i}\right)}^{2}}$$

where $y_{i}$ is the manual reference measurement, ${\hat{y}}_{i}$ is the measurement or prediction from the automated system, $\overset{-}{y}$ is the mean of the reference values, and n is the sample size.

The accuracy of all body dimension measurements and weight predictions was evaluated using the above metrics on the entire dataset (n = 30). The performance of different formulas for weight prediction was also compared based on these metrics. The repeatability and consistency of the system measurements were assessed by calculating the Intraclass Correlation Coefficient (ICC). All statistical analyses were performed using the Scipy and Scikit-learn libraries in Python 3.12.3.

## 3. Results

### 3.1. Accuracy Assessment of Automated Body Dimension Measurements

**Figure 5:**
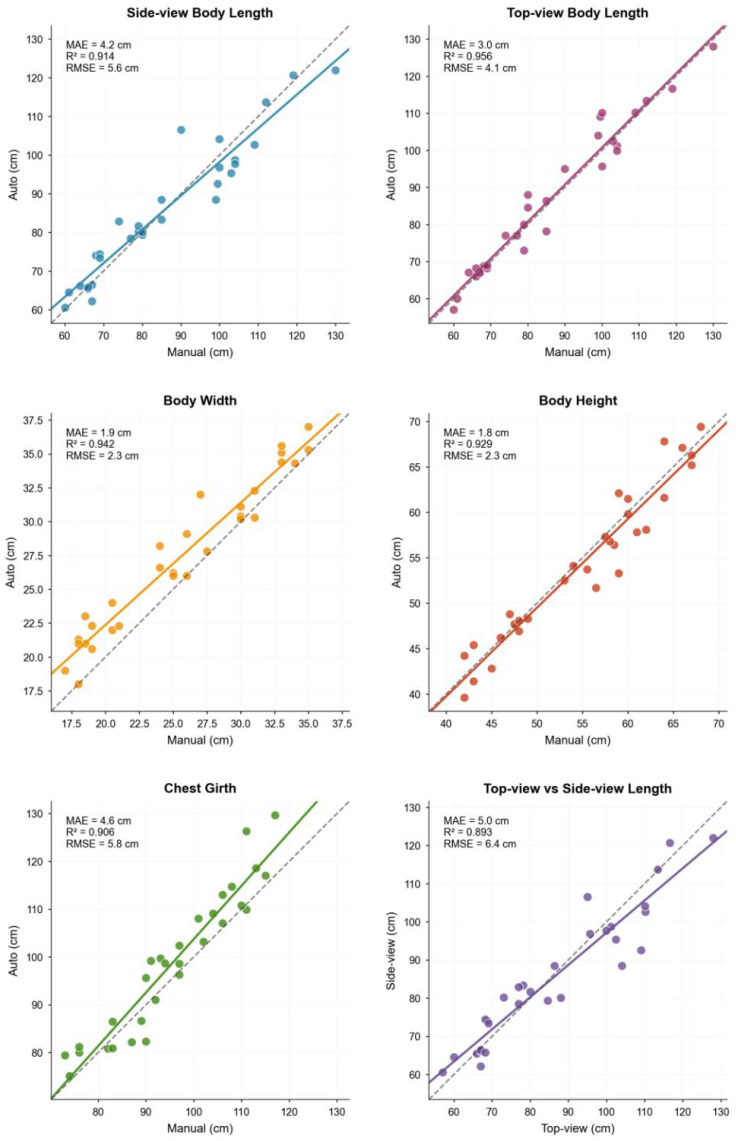
Comparison between automated and manual measurements of pig body dimensions.

To validate the precision of the proposed machine vision-based system for automated pig body dimension measurement, the results were rigorously compared against manual measurements as the ground truth. The evaluation encompassed four key parameters: body length, body width, body height, and chest girth. The comparative results are presented in Figure 5.

The automated measurement results exhibited a high degree of consistency with the manual body dimension measurements (R^2^ > 0.9) (Table 1):

The top-down view demonstrated optimal body length accuracy (MAE = 3.0 cm, RMSE = 4.1 cm, (R^2^ = 0.956)), outperforming the side-view body length measurements (MAE = 4.2 cm, RMSE = 5.6 cm, (R^2^ = 0.914)). This indicates that the top-down perspective minimizes occlusions and provides a more complete representation.

Body width (MAE = 1.9 cm, (R^2^ = 0.942)) and body height (MAE = 1.8 cm, (R^2^ = 0.929)) demonstrated high measurement accuracy, validating the system’s reliability;

Chest Girth (MAE = 4.6 cm, (R^2^ = 0.906)) achieved acceptable precision, laying the foundation for body weight modeling.

In summary, the machine vision-based automated measurement method has the capacity to effectively replace traditional manual techniques for the measurement of most body dimensions. The top-view technique in particular has distinct advantages for the capture of key parameters such as body length.

### 3.2. Performance Comparison of Chest Girth Fitting Algorithms

**Figure 6:**
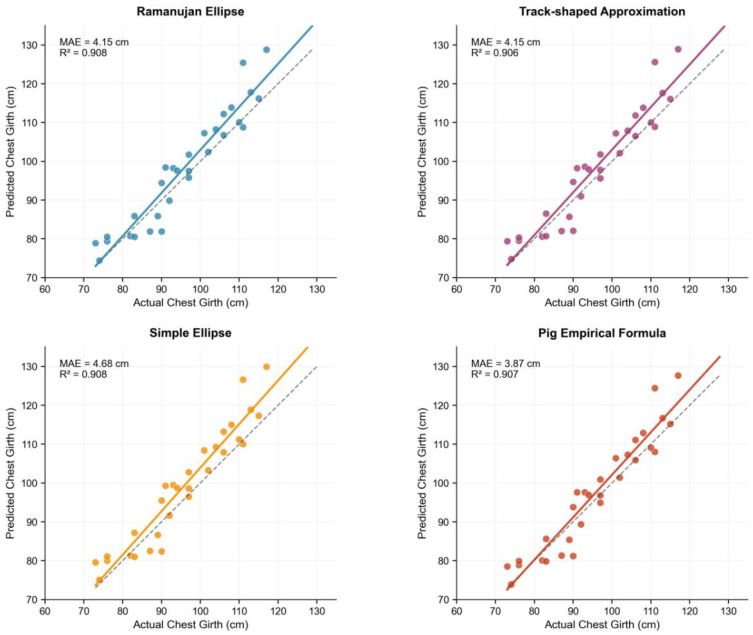
Performance comparison of different chest girth fitting algorithms.

Chest girth is a critical parameter for body weight estimation. Based on the elliptical cross-section defined by the body width (from top-view) and chest depth (from side-view), the performance of four distinct fitting algorithms for estimating chest girth was evaluated, as shown in Figure 6.

All four chest-girth proxies exhibited strong linear agreement with the actual body weight of the thirty pigs (R^2^ ≈ 0.957). The resulting prediction errors are summarized in Table 2.

Ramanujan’s elliptic approximation yielded the highest coefficient of determination and the lowest mean absolute error (MAE) and root mean square error (RMSE), with a mean deviation of only 3.24 kg—approximately 6% of the average body weight. Despite the pig-specific empirical formula producing a marginally smaller MAE (3.87 cm) in the chest-girth calibration demonstrated in Figure 6, the same algorithm regressed against actual body weight consistently ranked behind Ramanujan’s model. Consequently, owing to its superior accuracy, mathematical robustness, and broad applicability, the Ramanujan ellipse (MAE = 4.15 cm, R^2^ = 0.908) was adopted as the default chest-girth model for all subsequent body-weight predictions.

### 3.3. Development and Validation of Body Weight Estimation Models

To establish a highly accurate model for estimating pig body weight, multiple estimation formulas based on the automatically extracted body dimensions were developed and compared.

**Figure 7:**
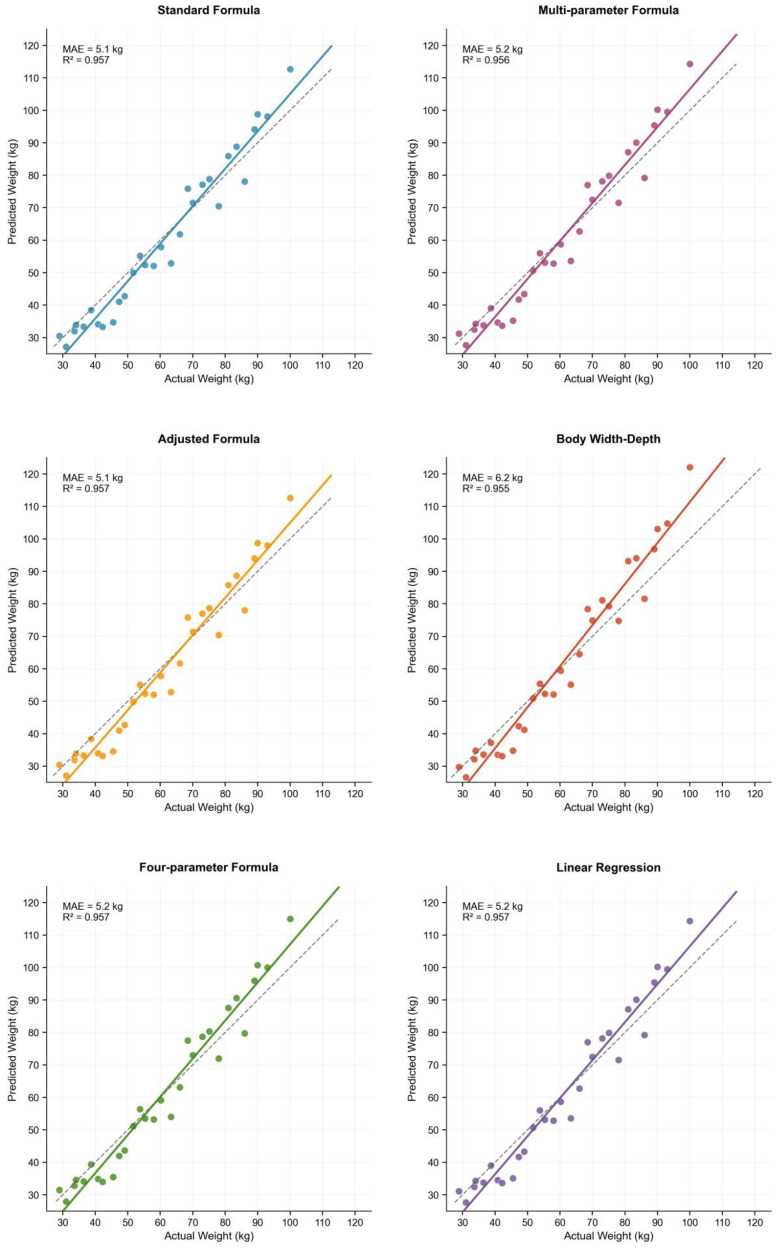
Performance comparison of different body weight estimation formulas.

As illustrated in Figure 7, the predicted body weights from all six models showed a strong correlation with the actual measured weights, with overall R^2^ values exceeding 0.955 and MAE ranging from 5.1 kg to 6.2 kg.

The Standard Formula (typically of the form: Weight = (Chest Girth)^2^ × Body Length/k) achieved the best overall performance (MAE = 5.1 kg, R^2^ = 0.957).

The Body Width-Depth model exhibited the lowest relative accuracy (MAE = 6.2 kg), suggesting that relying solely on cross-sectional parameters is insufficient for optimal weight estimation, and body length is an indispensable variable.

The Linear Regression model, Multi-parameter formula, and Four-parameter formula performed comparably to the Standard Formula. However, the Standard Formula was selected as the final model due to its simplicity, clear physical interpretability, and superior accuracy.

**Figure 8:**
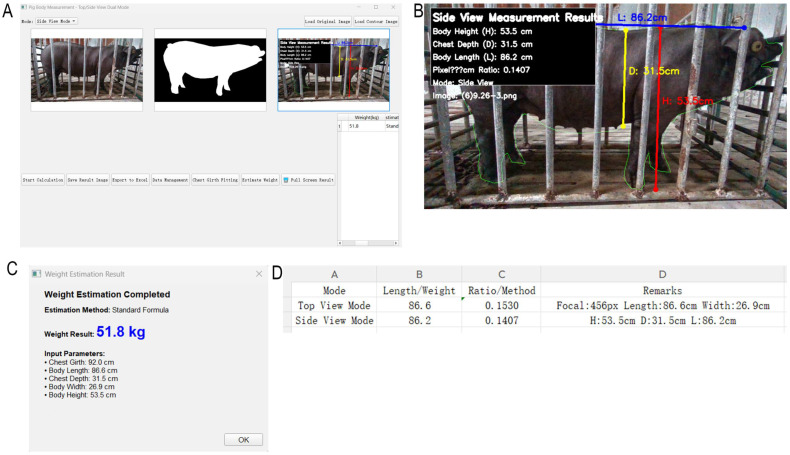
Interface of the automated pig body weight estimation system. (**A**) is the software interface diagram, (**B**) is the enlarged result diagram, (**C**) is the body weight fitting result diagram, (**D**) is the machine-measured body measurement data.

Consequently, the Standard Formula was adopted as the core algorithm and was further calibrated using linear regression based on 30 sets of actual weighing data to mitigate systematic bias. The calibrated model demonstrated reliable performance in practical application, as shown in the system interface in Figure 8.

This interface intuitively displays the automatically measured body dimensions (Body Length: 86.6 cm, Body Width: 26.9 cm, etc.), the chest girth (92 cm) fitted using Ramanujan’s approximation, and the final estimated body weight of 51.8 kg calculated by the calibrated Standard Formula.

This study successfully developed and validated an end-to-end machine vision system that automatically estimates pig body weight with high accuracy (R^2^ > 0.95) by leveraging optimized body dimension measurements, most notably through Ramanujan’s approximation for chest girth fitting.

## 4. Discussion

### 4.1. Core Performance Advantages of the Dual-View Depth Vision Measurement System

The “top-view + side-view” dual-view depth vision non-contact body measurement system for Jiangquan Black pigs proposed in this study demonstrates outstanding core performance, fully meeting the precision farming needs of small and medium-sized pig farms. With respect to body measurement, the determination coefficients (R^2^) for the four key parameters—body length, body width, body height, and chest depth—exceed 0.9. Among these, the top-view body length measurement demonstrates optimal accuracy (R^2^ = 0.956, MAE = 3.0 cm), while the body width (MAE = 1.9 cm) and body height (MAE = 1.8 cm) measurements exhibit superiority over the majority of existing solutions. For instance, Pezzuolo et al. [28] achieved an R^2^ of 0.839 for body length measurement using a Kinect v1-based system. Conversely, the system under consideration utilizes depth map calibration and dynamic scaling technology to effectively reduce perspective distortion and environmental noise, thereby significantly enhancing measurement stability. The Ramanujan ellipse perimeter model demonstrated superiority in predicting chest circumference and weight, with an MAE of 4.15 cm and an R^2^ of 0.908. The weight prediction model incorporating this chest parameter achieved an R^2^ of 0.957 and an MAE of 5.1 kg. However, it should be noted that the sample size used for weight calibration in this study was relatively small (n = 30), which may limit the model’s generalizability. In comparison to the point cloud mesh reconstruction model (error 4.89 kg) proposed by Kwon et al. [29] and the handheld RGB-D camera approach (MAE ≈ 9.25 kg) developed by Nguyen et al. [30], this system exhibits superior accuracy while circumventing the substantial computational demands of intricate point cloud reconstruction. This attribute renders it particularly well-suited for real-time monitoring in pig farms. Additionally, the system is capable of performing a single measurement in a mere 24 ± 4 s, which is more than 10 times faster than conventional manual methods. This efficiency is achieved without the need for physical restraint of the animals, thereby ensuring compliance with animal welfare standards. The hardware configuration consists of a single Intel RealSense D455 depth camera, with a depth error of no more than 2 mm, connected to a standard laptop computer. This configuration reduces hardware costs by 50% to 70% compared to multi-camera multi-view systems [31]. This approach effectively addresses the high-cost equipment adaptation challenge for small-to-medium-sized pig farms.

### 4.2. Key Technological Innovations and Principal Validation

The technological innovations of this study focus on the deep integration of multiple methods and engineering adaptation, with three core breakthroughs: first, optimizing the dual-view collaborative acquisition strategy by precisely positioning the top view (140 cm vertical height from the measurement area center) and side view (70 cm horizontal distance from the side edge) for full coverage of key body measurement regions—the top view suits horizontal measurements (e.g., body length/width) to avoid side-view pose distortion, while the side view uses a high-precision U-Net semantic segmentation network to accurately extract pig body core features and suppress background interference (fences, ground, etc.), verifying the “top view + side view” combination’s complementary advantages with performance significantly superior to single-view and redundant multi-view schemes [32]; second, integrating elliptical modeling with classical empirical formulas—modeling the pig’s torso cross-section as an ellipse (top-view body width as major axis, side-view chest depth as minor axis) and calculating chest circumference via Ramanujan’s formula, which solves non-contact measurement challenges while ensuring physical interpretability; compared with deep learning regression models relying on massive annotated data [20], the system BW_1_-based integrated empirical formula offers higher computational efficiency and simpler deployment, adapting to pig farms limited computing resources; third, simplifying efficient preprocessing—converting depth maps to metric units and replacing traditional background removal/binarization with U-Net semantic segmentation, greatly reducing complexity and environmental sensitivity; compared with conventional multi-step workflows [30], its preprocessing success rate exceeds 95%, delivering stronger robustness in real pig farm scenarios (uneven lighting, minor occlusions).

### 4.3. Comparison with Existing Research

In comparison with extant non-contact pig measurement studies, the primary advantage of this study lies in its three-dimensional balanced design of “precision + low cost + practicality”. The study focuses on Jiangquan Black Pigs and conducts specialized parameter fitting and calibration for their unique morphological characteristics (e.g., torso width-depth ratio, correlation between chest depth and body weight). In comparison with generic measurement solutions, this approach enhances breed adaptability, significantly reduces accuracy deviations caused by breed-specific traits, and provides a reference for measurement adaptation in pigs with similar body types. In principle, rather than adopting complex deep learning models without modification, high-precision measurement is achieved through the optimized integration of elliptical modeling, empirical formulas and semantic segmentation. This approach effectively mitigates accuracy losses due to breed variations and realizes “uncompromised precision with controllable costs” a distinction from studies relying on large neural networks [33]. In terms of application, existing multi-view systems [23] are mainly designed for large-scale pig farms requiring facility modifications. In contrast, this system can be directly deployed at pig farm exit passages without additional infrastructure investment, better aligning with the operational needs of small-to-medium-sized pig farms.

### 4.4. Future Research Directions and Application Prospects

It is proposed that future development be advanced in three aspects, building on existing research. It is imperative to introduce pose correction algorithms via keypoint detection (e.g., head, shoulders) for non-standard postures, and to integrate multi-frame image stitching to fill depth map holes, thus enhancing robustness in complex environments. The functionality of the system is such that it is able to extract texture and contour parameters from dual-view images. These are then combined with machine learning in order to construct a multi-indicator evaluation system. This system incorporates body condition scoring and backfat thickness estimation, and provides data support for precision feeding and early disease warning [34]. From a scenic perspective, the development of portable measurement terminals for free-range scenarios is imperative, in addition to the establishment of a cloud-based data management platform. The purpose of the former is to facilitate big data applications in regional swine farming, whilst the latter will enable the effective management of data. The system’s implementation will promote refined management (precision feeding, scientific breeding, early disease diagnosis) in small-to-medium-sized pig farms, driving the industry’s transformation from “experience-driven” to “data-driven” practices and aligning with the development trend of Precision Livestock Farming (PLF).

## 5. Conclusions

The present study aims to address the two core challenges of inefficient manual measurements and stress induction in small-to-medium-sized pig farms, as well as the high costs and limited practicality of existing automated systems. The development and validation of a non-contact body length and weight measurement system for Jiangquan Black pigs is based on dual-view depth vision (Intel RealSense D455). The system has been developed in alignment with the principles of Precision Livestock Farming (PLF) and welfare-oriented production, with the objective of achieving a balanced integration of accuracy, cost-effectiveness and user-friendliness. The integration of dual-view depth sensing with elliptical modeling establishes a replicable non-contact measurement paradigm for livestock.

With regard to the system’s performance, it is notable that it achieves a high level of accuracy in the measurement of key body dimensions, including length and width. The model’s core indicators demonstrate outstanding model fit and prediction reliability with physical interpretability, thus overcoming the challenge of balancing model complexity with practical applicability in livestock measurement. The U-Net semantic segmentation technology has been demonstrated to be effective in suppressing interference from complex farm backgrounds and enhancing environmental adaptability. The dual-camera configuration has been developed for utilization in small-to-medium-sized pig farms, with no requirement for facility modifications or adaptations. The configuration operates using standard laptops. The low hardware costs and absence of animal stress, in conjunction with pre-set parameter templates and simplified calibration steps, serve to significantly reduce the operational barriers for non-technical personnel.

Despite the system’s sensitivity to variations in pig posture, environmental obstructions, and breed differences, and its potential for segmentation errors under extreme lighting conditions, it validates the methodological value of integrating dual-view depth vision with elliptical modeling. This provides a scalable solution for non-contact measurement in other livestock and poultry. Future enhancements—including optimized deployment, improved algorithmic robustness against interference, and expanded functional modules (e.g., body condition scoring, backfat thickness estimation)—will further strengthen its suitability for smart livestock management, accelerating the industry’s transition towards data-driven, welfare-oriented precision farming.

## Figures and Tables

**Table 1:** Performance Comparison of Dimensional Measurements between Automated and Manual Methods for Pig Body Dimensions (n = 30).

Measurement Dimension	Mean Absolute Error (MAE, cm)	Root Mean Square Error (RMSE, cm)	Coefficient of Determination (R^2^)
Side-view Body Length	4.2	5.6	0.914
Top-view Body Length	3.0	4.1	0.956
Body Width	1.9	2.3	0.942
Body Height	1.8	2.3	0.929
Chest Girth	4.6	5.8	0.906
Top-view vs. Side-view Body Length	5.0	6.4	0.893

**Table 2:** Fitting performance against reference body weight (n = 30).

Model	MAE (kg)	RMSE (kg)	MAPE (%)	R^2^
Ramanujan ellipse	3.24	4.20	6.33	0.9597
Track-shaped approximation	3.39	4.29	6.33	0.956
Simple ellipse	3.39	4.33	6.33	0.9572
Pig-specific empirical formula	3.39	4.33	6.33	0.9577

## Data Availability

The raw data supporting the conclusions of this article will be made available by the authors on request.
